# Supplementary material for: An open-source framework for large-scale, flexible evaluation of biomedical text mining systems
Source: J Biomed Discov Collab. 2008 Jan 29;3:1. doi: 10.1186/1747-5333-3-1 (PMC2276192; doi:10.1186/1747-5333-3-1)
Supplement: Additional file 1 — Evaluation platform source code. The additional file contains the software evaluation framework discussed in this paper. The most current release can be downloaded from SourceForge.net [1]. See the accompanying README file for instructions on installation and use. Also included in the distribution is a collection of UIMA wrappers for some commonly used BioNLP tools and annotated corpora. [file 1747-5333-3-1-S1.zip › bionlp_uima_repository-1.0.1/docs/api/edu/uchsc/ccp/util/nlp/mention/SlotMention.html]

SlotMention (Center for Computational Pharmacology Util/UIMA API)


|  |  |  |
| --- | --- | --- |
| **Overview** \| **Package** \| **Class** \| **Tree** \| **Deprecated** \| **Index** \| **Help** | |  |
| **PREV CLASS**   **NEXT CLASS** | **FRAMES**    **NO FRAMES**     **All Classes** |  |
| SUMMARY: NESTED \| FIELD \| CONSTR \| METHOD | DETAIL: FIELD \| CONSTR \| METHOD |  |


---


## edu.uchsc.ccp.util.nlp.mention Class SlotMention

```
java.lang.Object
  edu.uchsc.ccp.util.nlp.mention.Mention
      edu.uchsc.ccp.util.nlp.mention.SlotMention
```

**All Implemented Interfaces:**: Comparable

---

``` public class SlotMention extends Mention ```

---

**Constructor Summary**

`SlotMention()`
  
          **Deprecated.**

`SlotMention(String mentionName)`


| **Method Summary** | |
| --- | --- |
| `void` | `addSlotValue(Object slotValue)`             Add a slot value to this slot mention |
| `int` | `compareTo(Object o)`             Compares two slot mentions by comparing their single line representations. |
| `boolean` | `equals(Object obj)`             Two slot mentions are equal if their slot mention name is equal, and their slot values are equal |
| `boolean` | `equals(SlotMention smToCompare, SlotMentionComparator slotMentionComparator, SpanComparator spanComparator, int maximumComparisonDepth, int depth)` |
| `String` | `getDocumentLevelSingleLineRepresentation()` |
| `Object` | `getFirstSlotValue()`             Return the first slot value |
| `String` | `getHashKey()`             **Deprecated.** |
| `String` | `getSingleLineRepresentation()`             Return a string representation of this SlotMention in a single line.  Representation: " [mentionName]:value1:value2:value3..." |
| `List<Object>` | `getSlotValues()`             Get the slot values |
| `int` | `hashCode()` |
| `static void` | `printMention(SlotMention sm, PrintStream ps, int indentLevel)`             Output a description of this slot mention |
| `void` | `setFirstSlotValue(Object slotValue)`             Set the first slot value. |
| `void` | `setSlotValues(List<Object> slotValues)`             Set the slot values |

**Methods inherited from class edu.uchsc.ccp.util.nlp.mention.Mention**

`getMentionName, setMentionName`

**Methods inherited from class java.lang.Object**

`clone, finalize, getClass, notify, notifyAll, toString, wait, wait, wait`

**Constructor Detail**

### SlotMention

```
@Deprecated
public SlotMention()
```

:   **Deprecated.**

---


### SlotMention

```
public SlotMention(String mentionName)
```


**Method Detail**

### getSlotValues

```
public List<Object> getSlotValues()
```

:   Get the slot values

    :   **Returns:**

---


### setSlotValues

```
public void setSlotValues(List<Object> slotValues)
```

:   Set the slot values

    :   **Parameters:**: `slotValues` -

---


### getFirstSlotValue

```
public Object getFirstSlotValue()
```

:   Return the first slot value

    :   **Returns:**

---


### setFirstSlotValue

```
public void setFirstSlotValue(Object slotValue)
```

:   Set the first slot value. If there is already a slotValue(s) in the list, then reset the value, else add a slotValue.

    :   **Parameters:**: `slotValue` -

---


### addSlotValue

```
public void addSlotValue(Object slotValue)
```

:   Add a slot value to this slot mention

    :   **Parameters:**: `slotValue` -

---


### getSingleLineRepresentation

```
public String getSingleLineRepresentation()
```

:   Return a string representation of this SlotMention in a single line.  
    Representation: " [mentionName]:value1:value2:value3..."

    :   **Overrides:**: `getSingleLineRepresentation` in class `Mention`
    :   **Returns:**

---


### getDocumentLevelSingleLineRepresentation

```
public String getDocumentLevelSingleLineRepresentation()
```

:   **Overrides:**: `getDocumentLevelSingleLineRepresentation` in class `Mention`

---


### printMention

```
public static void printMention(SlotMention sm,
                                PrintStream ps,
                                int indentLevel)
```

:   Output a description of this slot mention

    :   **Parameters:**: `sm` -: `ps` -: `indentLevel` - - controls the size of the indent from the left

---


### getHashKey

```
@Deprecated
public String getHashKey()
```

:   **Deprecated.**

    :   **Returns:**

---


### compareTo

```
public int compareTo(Object o)
```

:   Compares two slot mentions by comparing their single line representations. By default we use the IdenticalSlotMentionComparator

    :   **Specified by:**: `compareTo` in interface `Comparable` **Overrides:**: `compareTo` in class `Mention`

---


### equals

```
public boolean equals(Object obj)
```

:   Two slot mentions are equal if their slot mention name is equal, and their slot values are equal

    :   **Overrides:**: `equals` in class `Object`

---


### equals

```
public boolean equals(SlotMention smToCompare,
                      SlotMentionComparator slotMentionComparator,
                      SpanComparator spanComparator,
                      int maximumComparisonDepth,
                      int depth)
```

---


### hashCode

```
public int hashCode()
```

:   **Overrides:**: `hashCode` in class `Object`


---


|  |  |  |
| --- | --- | --- |
| **Overview** \| **Package** \| **Class** \| **Tree** \| **Deprecated** \| **Index** \| **Help** | |  |
| **PREV CLASS**   **NEXT CLASS** | **FRAMES**    **NO FRAMES**     **All Classes** |  |
| SUMMARY: NESTED \| FIELD \| CONSTR \| METHOD | DETAIL: FIELD \| CONSTR \| METHOD |  |


---
